# Machine Learning-Based Prediction of Six-Minute Walk Distance in Children with Obesity

**DOI:** 10.3390/children13081043

**Published:** 2026-08-05

**Authors:** Emna Makni, Mohamed Elloumi, Achraf Ammar, Mehdi Ben Brahim, Younes Hachana

**Affiliations:** 1 Research Laboratory of Exercise Physiology and Pathophysiology (LR19ES09), Faculty of Medicine Ibn Al-Jazzar, University of Sousse, Sousse 4002, Tunisia; emakni08@gmail.com; 2 Sport Sciences and Diagnostics Research Group, College of Sciences and Humanities, Prince Sultan University, Riyadh 11586, Saudi Arabia; 3 Department of Training and Movement Science, Institute of Sport Science, Johannes Gutenberg-University Mainz, 55122 Mainz, Germany; acammar@uni-mainz.de; 4 Research Laboratory, Molecular Bases of Human Pathology (LR19ES13), Faculty of Medicine of Sfax, University of Sfax, Sfax 3000, Tunisia; 5 Department of Nutrition and Food Technology, School of Agriculture, The University of Jordan, Amman 11942, Jordan; 6 Research Laboratory (LR23JS01) “Sport Performance, Health & Society”, Higher Institute of Sport and Physical Education of Ksar Saïd, University of “La Manouba”, Manouba 2037, Tunisia

**Keywords:** functional exercise capacity, predictive modelling, anthropometry, pediatric obesity, explainable artificial intelligence

## Abstract

**Highlights:**

**What are the main findings?**

- Explainable machine learning model, especially XGBoost, outperformed the conventional linear regression in predicting six-minute walk distance (6MWD), as it can better model complex and non-linear relationships between anthropometric parameters in children with obesity.
- Age is the main predictor of 6MWD in all models, with sex-specific different patterns of body mass index, height and waist circumference.

**What are the implications of the main finding?**

- The use of SHAP-based explainable AI with machine learning offers can bypass the limitations of conventional linear models and provide a transparent and accurate approach for the assessment of functional exercise capacity in pediatric obesity.
- These newly derived ML-informed prediction equations are practical tools for field-based clinical assessment tailored to each subgroup, but external validation in large cohorts is required before routine implementation.

**Abstract:**

**Background**: The six-minute walk test (6MWT) assesses functional exercise capacity, but existing reference equations for children with obesity rely on traditional linear regression, potentially overlooking complex, non-linear relationships between anthropometric characteristics and functional exercise capacity. **Objective**: This study aimed to develop and internally validate machine-learning (ML) prediction models and preliminary prediction equations derived from explainable ML models for six-minute walk distance (6MWD) in Tunisian school-aged children with obesity and to compare their predictive performance with a conventional regression-based approach. **Methods**: We analyzed data from 236 school-aged children with obesity (104 females, 132 males; 6–12 years). Anthropometric measurements included body mass (BM), height, body mass index (BMI), waist circumference (WC), and hip circumference (HC). Five models were evaluated: linear regression, Ridge, Lasso, Elastic Net, and XGBoost. Performance was assessed using five-fold cross-validation and evaluated by the mean absolute error (MAE) and root mean square error (RMSE). Predictor importance was assessed using SHapley Additive exPlanation (SHAP) and Gini importance. **Results**: XGBoost achieved the best predictive performance, with the lowest MAE (17.4 ± 2.5 m in females and 19.5 ± 1.9 m in males) and RMSE (25.3 ± 3.1 m in females and 26.4 ± 2.2 m in males). Age was the strongest predictor across all models (SHAP: 54.2–62.0%; Gini importance: 0.52–0.69), followed by height and BMI. Sex-specific analyses indicated that, in females, age and BMI contributed ~80% to the cumulative SHAP analysis; whereas, in males, age, height, and WC were the primary factors. **Conclusions**: ML, particularly XGBoost, significantly improves 6MWD prediction in school-aged children with obesity compared with traditional linear regression. Explainable ML increases model interpretability by evaluating the relative importance of anthropometric predictors. These obesity-specific prediction models may better capture complex non-linear associations between anthropometrics and functional exercise capacity. These initial population-specific models should be validated in larger independent samples before routine use clinical or field settings.

## 1. Introduction

Obesity is a major public health problem worldwide, with a rapidly increasing prevalence and significant health consequences, especially in the Americas and within the Middle East/North Africa region, where rapid nutrition transition and urbanization are driving marked increases in childhood obesity [1,2]. In Tunisia, the rising prevalence of obesity among children and adolescents poses specific challenges, as lifestyle and dietary changes are associated with reduced walking capacity and impaired cardiorespiratory fitness in this population [3,4]. Children who are overweight or obese are more likely to engage in sedentary behaviours and lower-intensity activities, as well as participate in passive after-school activities than normal-weight peers [5]. Consequently, the use of validated field-based assessment tools is necessary to objectively evaluate functional exercise capacity and its limitations in children with obesity [6].

The evaluation of functional exercise capacity in individuals with obesity increasingly relies on field-based walking tests. In this regard, the six-minute walk test (6MWT) is a practical and ecologically valid measure of submaximal functional capacity that has been widely accepted [6]. The 6MWT provides an integrated assessment of submaximal performance by simultaneously engaging cardiovascular, respiratory, neuromuscular, and metabolic systems during sustained ambulation [7,8,9]. In general, children with obesity walk shorter distances during the 6MWT than their normal-weight peers reflecting the mechanical and metabolic burdens of excess body mass during submaximal ambulation [10,11,12,13]. Several reference equations have been developed to predict six-minute walk distance (6MWD). Importantly, most existing models have been derived from mixed or normal-weight cohorts, raising concerns regarding their applicability to pediatric populations with obesity. These models often overlook important predictors such as body mass, which is key determinant of functional exercise capacity in this population [10,11,12,13].

In an important effort to address this gap, Makni et al. [14] developed a prediction equation for 6MWD specifically for children with obesity. The authors employed a classical linear regression framework to identify key anthropometric predictors of walking performance and developed one of the first obesity-specific reference models for pediatric functional assessment.

Nevertheless, linear regression methods are inherently constrained by assumptions of linearity, additivity, and limited interaction among predictors. Such assumptions may not adequately represent the complex and potentially non-linear relationships between anthropometric characteristics and functional exercise capacity in children with obesity. In recent years, machine-learning (ML) regression approaches have emerged as potential alternatives in health and exercise sciences, offering greater flexibility to model non-linear effects, higher-order interactions, and multi-collinearity without strong parametric constraints [15,16,17,18]. More recently, explainable ML approaches have further enhanced the applicability of these methods by enabling not only accurate prediction but also the transparent interpretation of variable importance. Such approaches have successfully been applied in sports biomechanics to identify the variables contributing most strongly to complex movement patterns and performance outcomes, thereby improving model interpretability alongside predictive performance [19].

Despite their growing application in health and exercise sciences, machine learning regression methods remain underutilized in the development of prediction equations for field-based functional tests in pediatric obesity. To the best of our knowledge, no study has applied explainable ML regression approaches to predict six-minute walk distance (6MWD) in children with obesity from North Africa, a region characterized by distinct anthropometric and lifestyle profiles and a rapidly increasing prevalence of childhood obesity [2].

Therefore, this study aimed to develop and validate ML models and prediction equations based on explainable ML to predict 6MWD in school-aged children with obesity. Their predictive performance was compared with traditional linear regression, and explainable ML approaches were used to assess the relative contribution of anthropometric predictors. The findings are meant intended to provide insights into the assessment of functional exercise capacity in pediatric obesity research.

## 2. Materials and Methods

### 2.1. Study Design and Participants

A cross-sectional study was conducted on a sample of 236 (104 females and 132 males), aged 6 to 12 years were recruited from primary and secondary schools in the region of Sousse, Tunisia. Participants in the study were recruited from Jawhara and Riadh districts using a standard questionnaire, including on rural and urban children. The inclusion criterion was a body mass index (BMI) > 97th percentile according to International Obesity Task Force (IOTF) cut-off points [20]. Exclusion criteria included any medical history of cardiovascular, respiratory, or musculoskeletal disease that would impair performance on the 6MWT. All participants and their parents/legal guardians provided written informed consent after being informed about the risks and benefits of participating in the study. The study protocol was approved by the local Ethics Committee of the Faculty of Medicine of Sousse (MHESR-2005-074/14, approval date: 10 January 2025) in accordance with the Declaration of Helsinki. Based on an a priori sample size calculation using the formula for cross-sectional mean estimation [21], a minimum of 319 participants was required to estimate the 6MWD with a precision of ±10 m (SD = 91.08 m) at a 95% confidence level. However, the final sample comprised 236 participants, which was lower than this requirement. Nonetheless, this sample size exceeded the recommended minimum of 10–20 participants per predictor variable for regression analysis [22], with a ratio of approximately 47 participants per predictor (236/5 = 47.2) and provided adequate statistical power (>80%) for detecting medium to large effect sizes in regression models [23]. The current study builds on previous linear regression work by Makni et al. [14] using a new sample to apply and assess ML and explainable artificial intelligence methods.

### 2.2. Anthropometric Assessment

All measurements were taken by trained personnel following standardized protocols. Body mass (BM, kg) was measured to the nearest 0.1 kg using a calibrated digital scale (Seca, Wandsbek, Germany), with participants wearing light clothing. Height was measured to the nearest 0.1 cm using a wall-mounted stadiometer (Holtain Ltd., Crymych, UK). Body mass index (BMI) was calculated as BM/height^2^ (kg/^2^). Waist circumference (WC) and hip circumference (HC) were measured with a non-elastic tape at the midpoint between the lowest rib and the iliac crest, and at the level of the greatest gluteal protuberance, respectively [24]. All measurements were performed twice, and the mean value was used for analysis.

### 2.3. 6MWT

The 6MWT protocol was performed in accordance with the procedure previously described by our group [14] and the guidelines of the American Thoracic Society (ATS) [25]. Participants walked along a flat, straight 30 m corridor, marked every 3 m, and were instructed to cover as much distance as possible in six minutes. Standardized verbal encouragement was given every minute. The total distance walked (6MWD) was recorded in metres.

### 2.4. Statistical and Machine-Learning Analyses

Continuous variables were initially assessed for normality using the Shapiro–Wilk test. As several variables were not normally distributed, descriptive statistics are presented as median and interquartile range (IQR). Associations between 6MWD and anthropometric variables were examined using Spearman’s rank correlation coefficients (ρ). Correlation coefficients were reported with 95% confidence intervals obtained using bootstrap resampling with 1000 iterations. Statistical significance was set at *p* < 0.05.

To predict 6MWD, five supervised regression algorithms were developed and compared: linear regression, Ridge, Lasso, Elastic Net, and Extreme Gradient Boosting (XGBoost).

All candidate predictors were entered into each model, including age, body mass, height, body mass index (BMI), waist circumference (WC), and hip circumference (HC). No automated feature selection procedure was performed before model development, allowing each algorithm to determine the relative contribution of each predictor.

Prior to model development, a comprehensive ML pipeline was established to ensure methodological rigour and reproducibility. The dataset was first screened for missing values and implausible observations. No missing data were identified in the final dataset. Continuous variables were standardized using z-score transformation (mean = 0, standard deviation = 1) for the linear, Ridge, Lasso, and Elastic Net models. Standardization was estimated using the training data only and subsequently applied to corresponding validation data to avoid data leakage. XGBoost was fitted using the original predictor scales, as these methods are inherently insensitive to feature scaling.

Model performance was evaluated using five-fold cross-validation. The dataset was randomly partitioned into five approximately equal subsets. During each iteration, four subsets were used for model training and the remaining subset for validation, ensuring that each observation served once as validation data. A fixed random seed (seed = 42) was used to ensure reproducibility of data partitioning. During each iteration, four subsets were used for model training and the remaining subset for validation, ensuring that each observation served as validation data.

Hyperparameters for each ML algorithm were optimized using grid search within the cross-validation framework. For XGBoost, the hyperparameters were tuned solely within the training folds of the cross-validation to avoid any leakage of information from the validation data. The optimal hyperparameters identified were: n_estimators = 100, max_depth = 5, learning_rate = 0.1, subsample = 0.8, colsample_bytree = 0.8, and min_child_weight = 1. Ridge, Lasso, and Elastic Net models were fitted with default parameters for baseline comparisons. A detailed summary of the final model configurations is provided in Supplementary Table S1.

Model performance was calculated as the average across the five validation folds.

Predictive performance was quantified using mean absolute error (MAE) and root mean square error (RMSE). Lower MAE and RMSE values indicated superior predictive performance. The mean ± standard deviation of each performance metric across the five validation folds was reported for all models to facilitate comparison of predictive accuracy and robustness.

For model interpretation, feature importance for the final tree-based XGBoost model was quantified using Gini importance. To further improve model interpretation, SHapley Additive exPlanations (SHAP) were also computed. Importantly, SHAP and Gini importance values were calculated using the final optimized XGBoost model fitted to the complete dataset after the model selection and cross-validation process was completed. These importance measures were used exclusively for post hoc interpretation and did not influence model training, predictor selection, or the estimation of predictive performance. Mean absolute SHAP values were calculated to quantify the average contribution of each predictor to model predictions. Predictor rankings obtained from SHAP were compared with Spearman correlation coefficients and Gini importance to assess consistency across different importance metrics.

To identify the most influential variables, predictors were ranked according to their mean absolute SHAP values, and cumulative SHAP contribution curves were constructed. Following previous explainable artificial intelligence studies, an approximately 80% cumulative SHAP threshold was used as a pragmatic criterion to identify the subset of variables explaining most of the model importance. This threshold was used solely for model interpretation and exploratory variable selection rather than formal statistical inference.

As a secondary, exploratory step, the SHAP-selected predictors were used to derive preliminary linear and allometric prediction equations. It should be noted that these equations are not derived from the ML algorithm but are simplified approximations of the relationships that the XGBoost model found. They were generated using the standard linear and non-linear regressions on the full dataset to provide more interpretable, field-friendly tools. These equations are considered preliminary and population-specific, requiring external validation.

Separate models were developed for the overall sample, girls and boys, to investigate potential sex-specific differences in predictor importance and model performance. All ML analyses were conducted using Python version 3.9.7 with the scikit-learn (version 1.0.2), XGBoost (version 1.5.0), SHAP (version 0.40.0), and SciPy (version 1.7.1) libraries.

## 3. Results

The descriptive characteristics of the study population are presented in Table 1. The total sample (n = 236, girls: 104, boys: 132), had a median age of 9.0 years [7.3–11.0]. Sex-specific analyses showed similar age distributions across groups. Median 6MWD was significantly higher in males (535.0 m [465.8–575.0]) than females (497.5 m [422.0–560.0]), together with expected differences in anthropometric characteristics, particularly height and WC. Continuous variables are presented as medians and interquartile range (IQR; 25th–75th percentile) because they were not normally distributed.

### 3.1. Spearman Correlation Analysis

**Figure 1:**
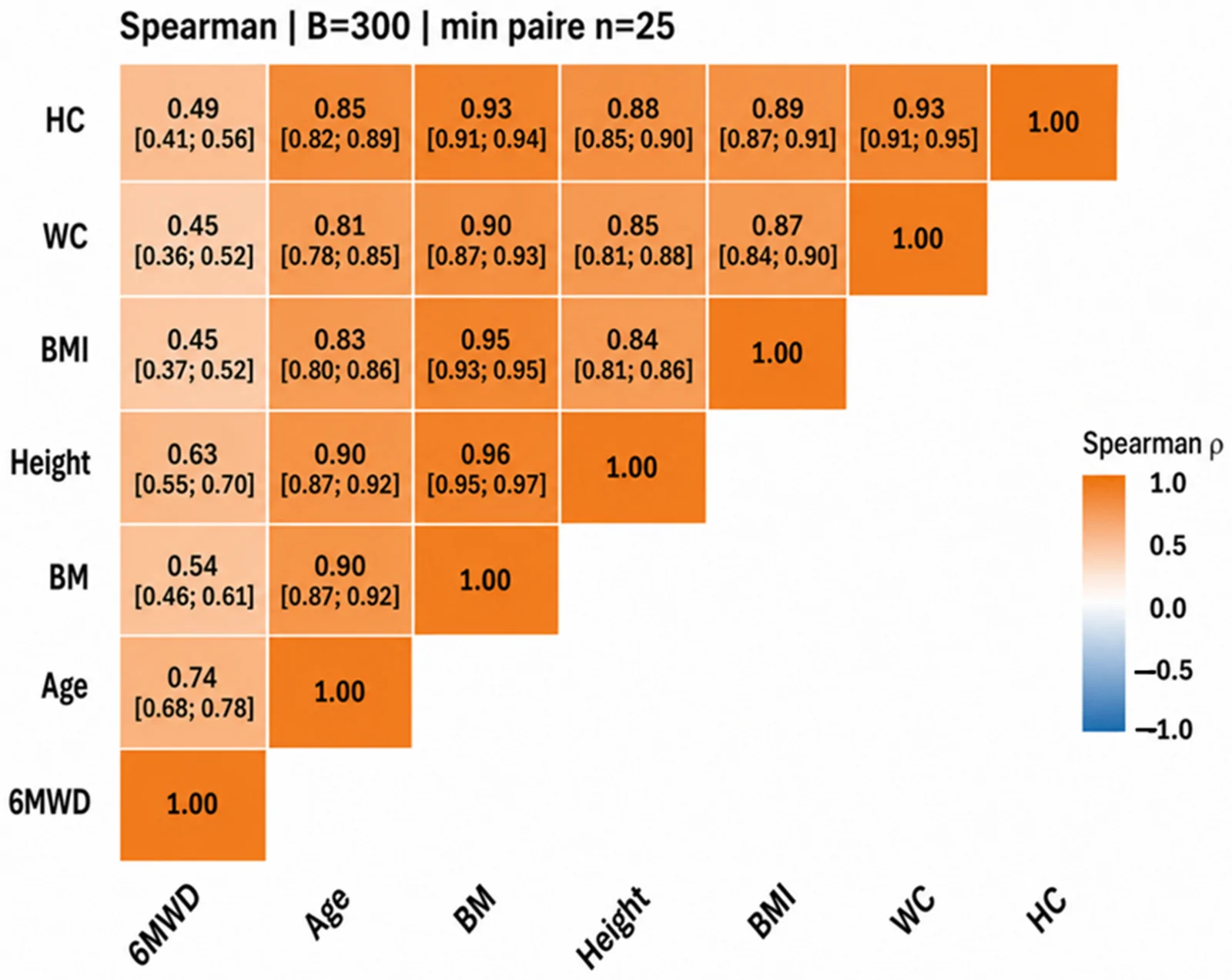
Heatmap of pairwise Spearman rank correlations (ρ [95% Confidence Intervals]) between 6MWD and anthropometric variables (N = 236). 6MWD: six-minute walk distance; BM: body mass; BMI: body mass index; HC: hip circumference; WC: waist circumference.

Spearman rank correlation analyses revealed significant associations between 6MWD and all predictors across the total sample and sex-specific subgroups (all *p* < 0.001). Age exhibited the strongest univariable correlation with 6MWD in all groups (|ρ| = 0.68–0.80), followed by stature and body mass-related indices (Figure 1).

In the total sample, 6MWD correlated strongly with age (ρ = 0.74) and moderately with height, BM, BMI, WC, and HC (ρ = 0.45–0.63), supporting their use in subsequent regression models.

### 3.2. Trained Models

**Figure 2:**
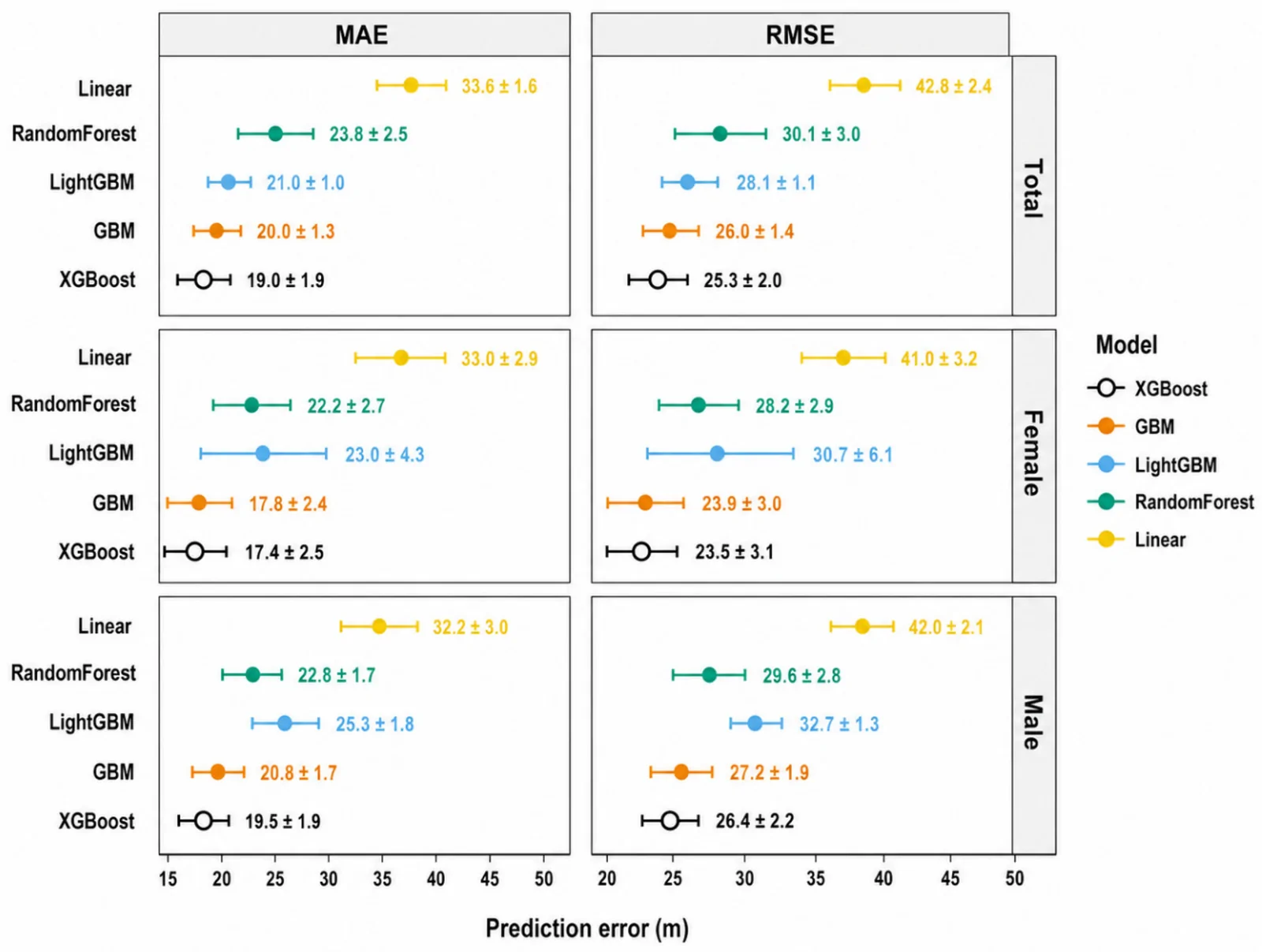
Five-fold cross-validated predictive performance of regression models for 6MWD. 6MWD: six-minute walk distance; CV: cross-validated; EN: Elastic Net; LR: linear regression; MAE: mean absolute error; RMSE: root mean square error; XGBoost: Extreme Gradient Boosting.

Five regression models were evaluated for their performance in predicting 6MWD: linear regression, Ridge, Lasso, Elastic Net, and XGBoost. The predictive performance obtained by five-fold cross-validation is summarized in Figure 2. In the overall sample, XGBoost achieved the lowest prediction error, with a mean MAE of 19.0 ± 1.9 m and an RMSE of 25.3 ± 2.0 m, outperforming linear regression (MAE 23.5 ± 1.6 m, RMSE 31.2 ± 2.1 m), Ridge Regression (MAE 23.2 ± 1.5 m, RMSE 30.8 ± 1.9 m), Lasso Regression (MAE 23.4 ± 1.6 m, RMSE 31.0 ± 2.0 m), and Elastic Net (MAE 23.3 ± 1.6 m, RMSE 30.9 ± 2.0 m). Similar findings were observed in sex-stratified analyses, with XGBoost yielding the lowest MAE in females (17.4 ± 2.5 m, RMSE 23.5 ± 3.1 m) and males (19.5 ± 1.9 m, RMSE 26.4 ± 2.2 m). Overall, XGBoost exhibited the best predictive performance in all analyses and was therefore selected as the final model for further interpretability analyses.

### 3.3. Feature Importance and Rank Consistency

Across all groups, age consistently emerged as the most influential feature, showing the highest absolute Spearman correlations (|ρ| = 0.68–0.80), the largest SHAP importance values (54.20–62.00), and the highest GINI importance scores (0.52–0.69), with high rank consistency across metrics. Height also showed high rank consistency in males (|ρ| = 0.68; SHAP = 19.7; GINI = 0.17) and in the total sample (|ρ| = 0.63; SHAP = 14.7; GINI = 0.20). In contrast, height showed lower rank consistency in females, despite a relatively strong correlation (|ρ| = 0.57). Other anthropometric variables exhibited moderate and group-specific rank consistency. In females, BMI showed moderate consistency (|ρ| = 0.37; SHAP = 17.2; GINI = 0.10), while BM, HC, and WC displayed weaker alignment between correlation- and model-based metrics. In males, BM, BMI, and HC showed moderate consistency (|ρ| = 0.43–0.62; SHAP = 13.2–15.9; GINI = 0.06–0.12); whereas, WC showed low consistency. In the total sample, BMI, BM, WC, and HC exhibited moderate consistency, with lower GINI and SHAP values than age and height (Table 2).

### 3.4. Cumulative SHAP Contribution and Selection of Dominant Predictors

**Figure 3:**
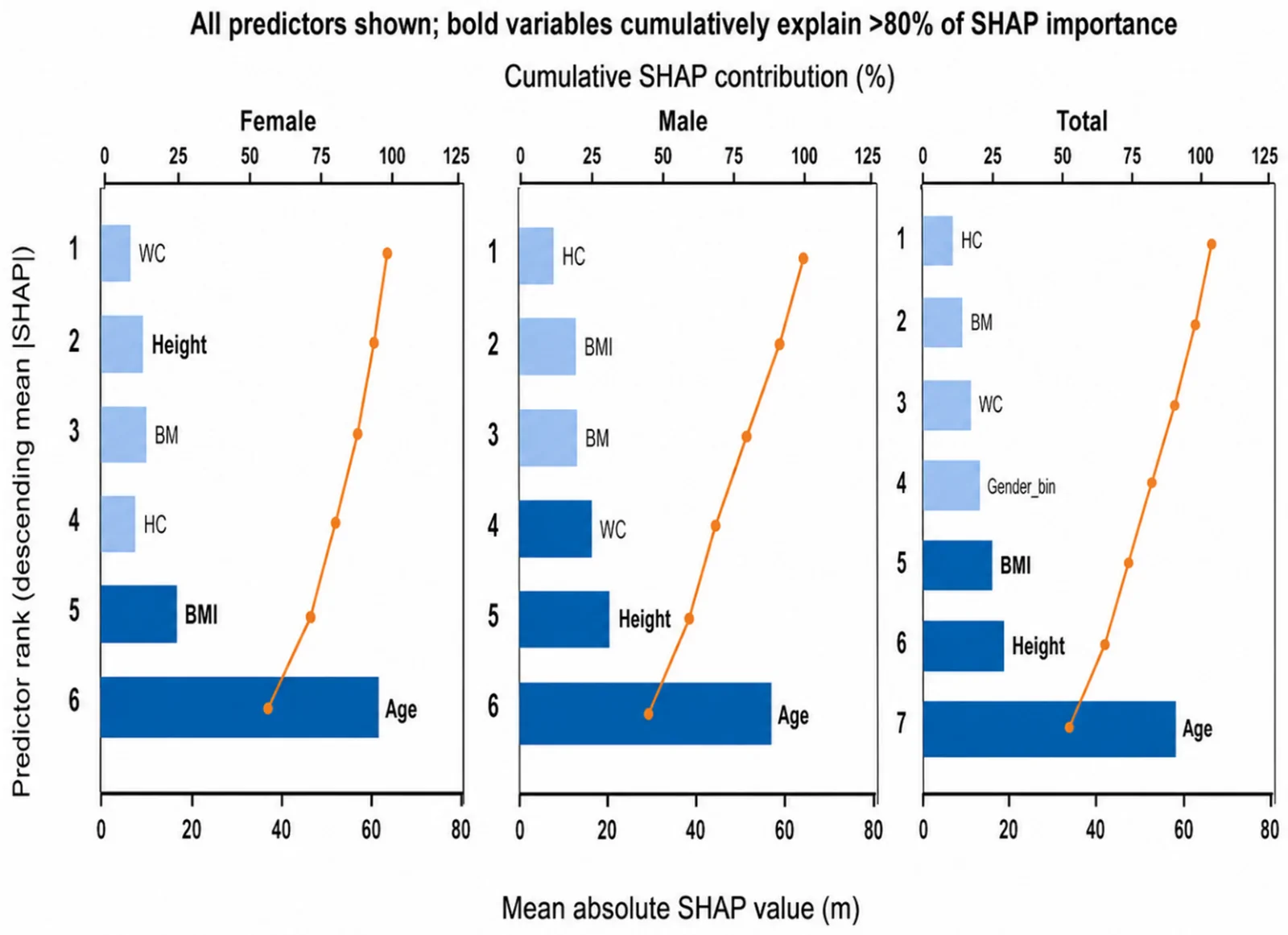
Cumulative SHAP contribution curves identifying the subset of predictors accounting for approximately 80% of total model importance in the overall sample and sex-specific subgroups. The orange line represents the cumulative percent-age of total SHAP importance as predictors are added in order of their individual importance. The dashed horizontal line marks the 80% threshold. BM: body mass; BMI: body mass index; HC: hip circumference; SHAP: SHapley Additive exPlanations; WC: waist circumference.

Cumulative SHAP contribution curves (Figure 3) showed that the predictors required to reach approximately 80% of total SHAP-based model importance differed between the total sample and sex-specific subgroups, with age as the only common predictor. In the total sample, age, height, and BMI together accounted for approximately 80% of the cumulative SHAP contribution. In females, this threshold was reached by age and BMI (~81%). In males, age, height, and WC captured approximately 89% of the total SHAP contribution.

## 4. Discussion

This study aimed to identify and rank the key anthropometric predictors of 6MWD in Tunisian children with obesity using explainable ML regressions. Among the five regression models evaluated in this study, XGBoost achieved the best predictive performance. However, because the differences between models were relatively small and no external validation was performed, this finding should be interpreted as evidence of better internal predictive performance rather than definitive superiority. SHAP analysis consistently identified age as the most influential predictor of 6MWD in both the overall sample and the sex-specific subgroups. Age explained the largest proportion of model importance across all analyses; whereas, the relative contributions of the remaining anthropometric variables differed between females and males. In the overall sample, age was followed by height and BMI. Age and BMI explained approximately 80% of the cumulative SHAP contribution in females, while in males the most important predictors were age, height, and WC.

Previous studies [14,26,27] have consistently identified anthropometric characteristics as important predictors of 6MWD in children, and the present findings broadly support this view. More specifically, age emerged as the variable most strongly associated with 6MWD in the total sample as well as in the female and male subgroups (Table 2), reinforcing the central role of growth-related development in walking performance. This age-related improvement pattern is consistent with the non-linear developmental trajectory reported across childhood and adolescence [28,29,30], indicating the gradual maturation of the musculoskeletal, cardiovascular, and neuromuscular systems. One of these studies examined change in direction performance rather than 6MWD, but both outcomes show improvements with age related to growth and maturation.

An additional finding was that in bivariate analysis anthropometric variables such as body mass, WC, HC, and BMI were positively correlated with 6MWD, but their relative importance decreased after multivariable ML modelling, suggesting that the initial positive relationships were primarily influenced by normal growth process. In practical terms, larger body dimensions do not necessarily translate into better walking performance in youth; instead, for children of similar age and height, increased body mass and central adiposity are associated with poorer 6MWD. This pattern is compatible with a suppression effect; whereby bivariate associations mainly reflect shared developmental trends rather than direct effects of body composition on performance. In children, functional capacity depends on chronological age and physical characteristics, but also on biological age and maturation. In a study of children aged 6 to 12 years with obesity, maturation differences may have influenced fat distribution and biological maturity among participants, which could have led to sex-specific differences in the importance of predictors in the model presented. In addition, personal motivation during the performance tests is an important factor affecting in the 6MWD outcomes, although it was not assessed quantitatively. While verbal encouragement was standardized, individual engagement may vary and thus affect performance. This highlights association between physiological capacity and psychological factors in children, particularly motivation, which has been demonstrated to influence 6MWD [31]. Traditional linear models have difficulty representing these non-linear growth spurts and maturation changes in body composition, which involve complex interaction, highlighting the need for ML techniques in pediatric populations.

From a statistical and methodological perspective, the transition from conventional predictive analyses to ML approaches reflects a shift away from models constrained by pre-specified functional forms and assumptions of largely linear, additive, and independent effects, toward more flexible frameworks better suited to capturing non-linear associations, higher-order interactions, and the complex dependency structures that often characterize biological and performance-related data [32]. More recently, explainable ML frameworks have further enhanced these approaches by combining high predictive performance with transparent model interpretation, allowing researchers to identify the variables contributing most strongly to complex biomechanical and performance-related outcomes. The present methodological approach is consistent with previous applications of explainable ML techniques in sports biomechanics, where tree-based ensemble models combined with model interpretation methods successfully characterized complex movement–performance relationships while improving model interpretability [19]. In the present study, five ML regression models were trained for predicting 6MWD. Among the five evaluated regression models, XGBoost achieved the best predictive performance and was therefore selected as the final model for subsequent interpretation using SHAP and Gini importance [33]. From a clinical perspective, the XGBoost model resulted in the lowest prediction errors, with a 4–5 m reduction in MAE and RMES as compared with linear regression. This difference, while unlikely to alter individual decision-making, indicates that ML can capture non-linear anthropometric relationships overlooked by linear models. The increase in complexity of the ML approaches is thus not justified by a significant gain in predictive performance, but by the increased interpretability and the possibility of discovering complex physiological patterns specific to subgroups by SHAP analysis, providing more personalized clinical insights.

The cumulative SHAP analysis was used to provide an understanding of how a reduced set of variables could summarize the predictive structure of a trained model [34]. SHAP values can be aggregated to derive cumulative contributions, allowing the identification of the most influential predictors in a trained model. In practice, a threshold is often adopted as a pragmatic convention to facilitate interpretation and guide variable selection [35]. In the present study, an 80% cumulative SHAP threshold was applied within the trained XGBoost model, as a pragmatic criterion to identify the most influential predictors of 6MWD. Moreover, anthropometric characteristics were the only predictors included in the analysis of the present study. However, lifestyle and activity-related behaviours are important determinants of physical fitness and cardiorespiratory fitness in young people. The importance of adhering to the 24 h movement guidelines, which include physical activity, sedentary behaviour and sleep, on the functional capacity of children is well recognized [35]. The inclusion of these behavioural determinants, in addition to anthropometric characteristics, would allow future models to predict 6MWD to provide a more complete assessment of functional exercise capacity.

As a secondary and exploratory aim, the present study used SHAP-selected predictors to derive preliminary linear and allometric formulations for 6MWD prediction in children with obesity. These formulations were intended to provide simplified, interpretable representations of the patterns identified by the XGBoost model, rather than definitive or externally validated prediction equations. For the total sample, the linear equation was 6MWD = 186.4 + 38.1·age + 1.9·height − 2.1·BMI, with the allometric counterpart 6MWD = 112.3 × age^0.89^ × height^0.42^ × BMI^−0.18^; this model explained 68% of the variance (R^2^ = 0.68, Adj. R^2^ = 0.67), with a cross-validated MAE of 20.8 m and RMSE of 27.5 m. For females, the linear equation was 6MWD = 312.7 + 42.5·age − 2.9·BMI − 1.8·HC, with the allometric counterpart 6MWD = 245.6 × age^0.85^ × BMI^−0.22^ × HC^−0.34^; this model yielded an R^2^ of 0.65 (Adj. R^2^ = 0.63), a cross-validated MAE of 19.6 m, and an RMSE of 25.8 m. For males, the linear equation was 6MWD = 98.5 + 34.8·age + 2.2·height − 1.5·WC, with the allometric counterpart 6MWD = 87.4 × age^0.72^ × height^0.58^ × WC^−0.21^; this model produced an R^2^ of 0.70 (Adj. R^2^ = 0.69), a cross-validated MAE of 21.4 m, and an RMSE of 28.1 m. A full summary of these equations, including standard errors for all coefficients and cross-validated performance metrics, is provided in Supplementary Table S2. When considered together, the linear and allometric equations identified predictors that were broadly consistent with previous studies based on conventional modelling approaches, particularly the central role of age and the contribution of body-size characteristics [14,26]. However, the ML-based strategy used in the present study provided additional information by clarifying the relative importance of these predictors and by highlighting potential subgroup-specific patterns that may be less apparent in traditional models. In this sense, the XGBoost–SHAP framework should be viewed as complementary to, rather than a replacement for, conventional statistical modelling. The derived equations offer simplified and interpretable representations of the relationships captured by the ML models, but they should be interpreted cautiously. Although the combination of XGBoost, SHAP explainability, and cross-validated tuning strengthens the internal coherence of predictor selection, it does not fully overcome potential instability in regression coefficients, especially in the presence of correlated anthropometric variables. Differences between SHAP and Gini rankings for secondary predictors further suggest that variable importance may depend on the metric used and on the shared information among body-size measures. Therefore, the proposed equations should be regarded as preliminary, ML-informed formulations that are physiologically plausible and internally supported but still require confirmation in larger and independent samples before being considered stable or generalizable.

### Strength and Limitations

A strength of the present study is the combined use of conventional statistical analyses with explainable ML approaches, allowing both prediction and transparent interpretation of variable importance, an approach increasingly recommended in sports biomechanics and movement science [19]. However, several limitations should be acknowledged. First, the absence of external validation limits the generalizability of the present findings. Second, the total sample size at the end of the study (n = 236) was smaller than the calculated initial target. While the sample size was adequate for the primary predictors, it was limited for sex-specific subgroups and this could have impacted the stability of the SHAP estimates, the reproducibility of secondary predictors, and increased the risk of model overfitting. The implementation of five-fold cross-validation aimed to mitigate the risk enhance model robustness. Therefore, the relative importance of secondary variables should be interpreted with caution. Third, biological age, maturation status, and psychological factors such as personal motivation and engagement during the 6MWT were not considered in this study and may have influenced walking performance in children with obesity. Finally, the proposed linear and allometric equations should be considered as preliminary, population-specific approximations that require validation in larger and independent samples.

## 5. Conclusions

This study investigated the performance of ML approaches in predicting the 6MWD in school-aged children with obesity and identified the most influential predictors of this performance. Of the five models tested, XGBoost was the most suitable model for further analysis. SHAP-based interpretation revealed age as the most consistent predictor across all groups, while the relative contribution of the remaining anthropometric characteristics differed between the overall sample, females, and males. The complex ML output was converted into more interpretable subgroup-specific prediction equations (linear and allometric) using the predictors identified by SHAP. These findings suggest that ML-informed modelling may complement conventional regression approaches for identifying relevant predictors of 6MWD in children with obesity and for supporting the development of prediction equations. Nevertheless, the proposed equations should be interpreted as preliminary, population-specific prediction equations that require external validation in large, independent cohorts before they can be considered reference equations or recommended for clinical, field-based, or research applications.

## Figures and Tables

**Table 1:** Descriptive characteristics of the study population (median [IQR]).

Variable	Total (n = 236)	Females (n = 104)	Males (n = 132)
Age, y	9.0 [7.3–11.0]	9.0 [7.2–11.0]	9.0 [7.5–11.0]
Height, cm	134.0 [128.4–146.3]	130.0 [127.0–147.0]	135.5 [128.0–146.0]
BM, kg	38.0 [30.5–46.6]	38.2 [28.5–45.9]	37.5 [31.2–46.7]
BMI, kg·m^−2^	21.0 [19.5–22.5]	21.1 [19.4–23.2]	20.4 [19.5–22.0]
WC, cm	82.0 [75.0–87.0]	80.0 [71.8–85.3]	83.0 [75.8–90.0]
HC, cm	85.5 [79.0–93.3]	84.0 [78.0–90.0]	87.0 [80.0–98.0]
6MWD, m	520.0 [431.8–573.6]	497.5 [422.0–560.0]	535.0 [465.8–575.0]

BM: body mass; BMI: body mass index; 6MWD: six-minute walk distance.

**Table 2:** Feature importance and rank consistency across correlation and XGBoost-based metrics.

Group	Feature	Spearman |ρ|	SHAP Importance	GINI Importance
**Female**	Age	0.68	62.0	0.69
	Height	0.57	5.60	0.08
	BM	0.47	8.50	0.06
	BMI	0.37	17.2	0.10
	WC	0.47	4.00	0.03
	HC	0.45	9.50	0.05
**Male**	Age	0.80	54.20	0.52
	Height	0.68	19.70	0.17
	BM	0.62	13.50	0.12
	BMI	0.53	13.20	0.11
	WC	0.43	15.90	0.06
	HC	0.53	4.60	0.02
**Total**	Age	0.74	60.60	0.56
	Height	0.63	14.70	0.20
	BMI	0.45	13.40	0.09
	BM	0.54	7.10	0.06
	WC	0.45	8.70	0.04
	HC	0.49	7.00	0.04

BM: body mass; BMI: body mass index; GINI: Gini importance (feature importance based on node impurity reduction); HC: hip circumference; ρ: Spearman’s rank correlation coefficient; SHAP: SHapley Additive exPlanations; WC: waist circumference; |ρ|: absolute value of Spearman’s rank correlation coefficient.

## Data Availability

The data presented in this study are available on request from the corresponding author. The data are not publicly available due to ethical and confidentiality restrictions regarding research involving children, as imposed by the ethics committee and the terms of participant informed consent.

## Supplementary Materials

The following supporting information can be downloaded at: https://www.mdpi.com/article/10.3390/children13081043/s1, Table S1. Final hyperparameter settings for all regression models; Table S2. SHAP-informed linear and allometric prediction equations for 6MWD, with model fit statistics.
