# Supplementary material for: Machine Learning-Based Prediction of Six-Minute Walk Distance in Children with Obesity
Source: Children (Basel). 2026 Aug 5;13(8):1043. doi: 10.3390/children13081043 (PMC13511065; doi:10.3390/children13081043)
Supplement: Supplementary file 1 [file children-13-01043-s001.zip › children-4445658-supplementary.pdf]

Supplementary Table S1. Final hyperparameter settings for all regression models.

Algorithm	Hyperparameter	Value / Setting
Linear Regression	fit_intercept	True
Ridge	alpha	1.0
	solver	auto
Lasso	alpha	1.0
	max_iter	1000
	tol	0.0001
Elastic Net	alpha	1.0
	l1_ratio	0.5
	max_iter	1000
	tol	0.0001
XGBoost	n_estimators	100
	max_depth	5
	learning_rate	0.1
	subsample	0.8
	colsample_bytree	0.8
	min_child_weight	1
	objective	reg:squarederror
	eval_metric	rmse
	random_state	42

alpha: regularization strength parameter; auto: automatic solver selection; colsample_bytree: fraction of features used for each tree; eval_metric: evaluation metric; fit_intercept: whether to fit the intercept term; l1_ratio: Elastic Net mixing parameter (0 = Ridge, 1 = Lasso); learning_rate: step size shrinkage (XGBoost); max_depth: maximum tree depth; max_iter: maximum number of iterations; min_child_weight: minimum sum of instance weight in a child; n_estimators: number of boosting rounds; objective: loss function; random_state: random seed for reproducibility; rmse: root mean square error; solver: optimization algorithm; subsample: fraction of training data used for each tree; tol: tolerance for optimization.

Supplementary Table S2. SHAP-informed linear and allometric prediction equations for 6MWD, with model fit statistics.

Group	SHAP-selected predictors	Linear Equation (6MWD, m)	Allometric Form (6MWD, m)	R^2	Adj. R^2	CV-MAE (m)	CV-RMSE (m)
**Total**	Age (years), Height (cm), BMI (kg/m²)	6MWD = 186.4 + 38.1·age + 1.9·height − 2.1·BMI	$6MWD = 112.3 \times age^{0.89} \times height^{0.42} \times BMI^{-0.18}$	0.68	0.67	20.8	27.5
**Females**	Age (years), BMI (kg/m²), HC (cm)	6MWD = 312.7 + 42.5·age − 2.9·BMI − 1.8·HC	$6MWD = 245.6 \times age^{0.85} \times BMI^{-0.22} \times HC^{-0.34}$	0.65	0.63	19.6	25.8
**Males**	Age (years), Height (cm), WC (cm)	6MWD = 98.5 + 34.8·age + 2.2·height − 1.5·WC	$6MWD = 87.4 \times age^{0.72} \times height^{0.58} \times WC^{-0.21}$	0.73	0.72	19.5	26.4

6MWD: six-minute walk distance; BMI: body mass index; HC: hip circumference; WC: waist circumference; R^2: coefficient of determination; Adj. R^2: adjusted R^2; CV-MAE: cross-validated mean absolute error; CV-RMSE: cross-validated root means square error.
